# ZIF Nanocrystal-Based Surface Acoustic Wave (SAW) Electronic Nose to Detect Diabetes in Human Breath

**DOI:** 10.3390/bios9010004

**Published:** 2018-12-26

**Authors:** Fabio A. Bahos, Arianee Sainz-Vidal, Celia Sánchez-Pérez, José M. Saniger, Isabel Gràcia, María M. Saniger-Alba, Daniel Matatagui

**Affiliations:** 1 Instituto de Ciencias Aplicadas y Tecnología (ICAT), Universidad Nacional Autónoma de México, Ciudad Universitaria, Ciudad de México 04510, Mexico; fbahos@gmail.com (F.A.B.); arianee.sainz@ccadet.unam.mx (A.S.-V.); celia.sanchez@ccadet.unam.mx (C.S.-P.); jose.saniger@ccadet.unam.mx (J.M.S.); 2 Instituto de Microelectrónica de Barcelona (IMB), CSIC, Campus UAB, 08193 Bellaterra, Spain; isabel.gracia@imb-cnm.csic.es; 3 Instituto Nacional de la Nutrición Salvador Zubiran, Department of Neurophysiology, Tlalpan 14080, Mexico; mariadelmarsaniger@gmail.com; 4 SENSAVAN, Instituto de Tecnologías Físicas y de la Información (ITEFI), CSIC, Serrano 144, 28006 Madrid, Spain

**Keywords:** eNose, gas sensor, SAW, surface acoustic wave, Love wave, diabetes, breath, VOC, ZIF, Zeolite

## Abstract

In the present work, a novel, portable and innovative eNose composed of a surface acoustic wave (SAW) sensor array based on zeolitic imidazolate frameworks, ZIF-8 and ZIF-67 nanocrystals (pure and combined with gold nanoparticles), as sensitive layers has been tested as a non-invasive system to detect different disease markers, such as acetone, ethanol and ammonia, related to the diagnosis and control of diabetes mellitus through exhaled breath. The sensors have been prepared by spin coating, achieving continuous sensitive layers at the surface of the SAW device. Low concentrations (5 ppm, 10 ppm and 25 ppm) of the marker analytes were measured, obtaining high sensitivities, good reproducibility, short time response and fast signal recovery.

## 1. Introduction

One of the great challenges of contemporary science is the efficient diagnosis of diseases using non-invasive techniques. This strategy aims to provide a higher quality of life for humans and reduce the mortality rate. Additionally, early treatment of diseases and its complications has an important economic impact by helping to avoid or reduce treatment costs.

The last portion of deeply exhaled breath, representing alveolar air, can be considered the headspace gas of blood. Exhaled breath, recognized mainly through the sense of smell, is a method that has long been used for disease diagnosis. This method was abandoned due to the emergence of new accurate and effective techniques, despite being highly invasive. Over the last few decades, an important advance in gas analysis technologies has re-launched the idea of diagnosing diseases by analyzing exhaled breath. Various studies using these techniques, such as gas chromatography-mass spectrometry (GC-MS), proton transfer reaction-mass spectrometry (PTR-MS), selected ion flow tube-mass spectrometry (SIFT-MS), ion mobility and optical absorption [1,2,3,4,5], have shown a link between the chemical composition of exhaled breath and certain diseases. Chemical compounds present in exhaled breath that change due to diseases are known as markers. The conventional systems mentioned above are accurate but are also bulky, expensive, and require highly-qualified operators. This has created a demand for low-cost systems with high sensitivity and small dimensions based on solid-state chemical sensors, with different detection principles such as impedance [6], resistive [7,8], optical [9] and piezoelectric [10,11], surface acoustic wave (SAW), the last of which is one of the most sensitive piezoelectric devices [12]. The design of materials with advanced features led to a new generation of chemical sensors with enhanced sensitivity and response time [13,14,15]. Due to their unique porous structure zeolites have been used to detect gases [16]. However, in the last few years, organic zeolites such as zeolitic imidazolate frameworks (ZIFs) have attracted major attention as gas detectors [17,18,19,20,21], because they offer two primary advantages over conventional zeolites. First, they have larger pore sizes (about 1.16 nm for ZIF-8 and ZIF-67) and usually a smaller crystal size, resulting in higher surface area. Second, hydrophobic behavior is more pronounced in many ZIFs [22,23,24].

The World Health Organization (WHO) recognizes Diabetes mellitus, known as diabetes, as a serious and chronic disease that in 2012 caused 1.5 million deaths. A recent study reported in 2015 from 111 countries, estimated that there were 415 million people with diabetes aged 20–79 years, 5 million deaths attributable to diabetes, and the total global health expenditure due to diabetes was estimated to be 673 billion US dollars representing a substantial clinical and public health burden [8]. Moreover, the number of cases of diabetes among youths [25] and infancy [26] has increased in recent years but information on recent incidence trends is lacking and only statistical data for some countries are available. 

The presence of ketones in exhaled breath is a warning sign of ketosis that is related to fat catabolism either due to carbohydrate deprivation or its lack of utilization in persons with diabetes. This condition is known as diabetic ketoacidosis and requires immediate treatment. One type of ketone, known as acetone, provides a non-invasive measure of ketosis through breath. The basal level of acetone in a healthy people can be around 2 ppm [5,27]. Adults following low-carbohydrate diets can have elevated levels of ketones up to 40 ppm [28,29,30], and poorly controlled diabetes can cause ketoacidosis which can increase acetone concentration up to 1250 ppm [27,31]. However, human exhaled air is a complex mixture of chemical compounds, making the detection and stage classification of a determinate disease through a unique marker difficult, so that different disease markers need to be considered as indicators. Another marker related to blood glucose concentration and present in exhaled breath is ethanol, which is not directly produced by any known mammalian cellular biochemical pathway, and may increase in exhaled gas mixtures because of alcoholic fermentation of an excessive overload of carbohydrate-rich food in conjunction with overgrowth of intestinal bacteria. Ethanol used in combination with exhaled acetone allowed the prediction of fluctuating plasma glucose concentrations in a multi-linear regression model [32,33,34,35], demonstrating that it can be helpful in determining diabetes through exhaled breath. However, diabetes is the cause of half of the cases of renal failure. Kidney failure is related with ammonia levels higher than 3 ppm in exhaled breath [5,30,36,37]. Consequently, a finger print using breath levels of acetone, ethanol and ammonia could be a non-invasive predictor of diabetes, its control, and a proxy for damage caused by the disease.

In the present work, a SAW eNose, based on ZIF nanocrystals as sensitive layers, has been tested to detect acetone, ethanol, and ammonia as a potential non-invasive system to diabetes diagnosis and control.

## 2. Materials and Methods

### 2.1. Materials

All the reagents were purchased from a commercial provider (Sigma-Aldrich, St. Louis, MO, USA) and used without further purification, including ZnCl_2_ (98%), CoCl_2_·6H_2_O (98%), and 2methylimidazole (99%), hydrogen tetrachloroaurate (III) hydrate (HAuCl_4_, 99.9%) and trisodium citrate dihydrate (HOC (COONa) (CH_2_COONa)_2_ · 2H_2_O).

### 2.2. Synthesis of ZIF-8 and ZIF-67

**Figure 1:**
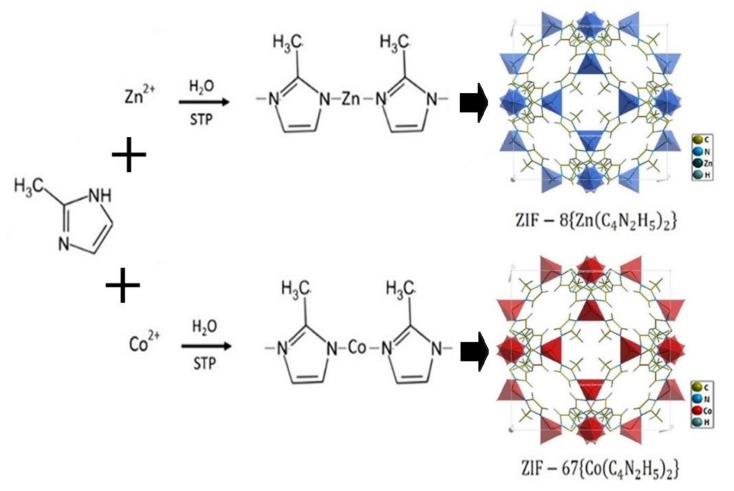
Representative synthesis and crystal structures of ZIF-8 and ZIF-67 used as the sensitive layers of gas sensors.

ZIF-8 and ZIF-67 samples were synthesized using the aqueous method reported elsewhere [38]. For ZIF-8 synthesis, a solution of 1.17 g of zinc chloride dissolved in 8 mL deionized (DI) water was added into a solution of 2-methylimidazole (2MeIM) (22.70 g) dissolved in 80 mL DI water, to yield a molar ratio of 2-methylimidazole to zinc of 70:1. The mixture was stirred at room temperature for 5 min. The product was collected by centrifugation (24,000 rpm, 10 min), washed in DI water three times and dried at 65 °C for 24 h in an oven. ZIF-67 was synthesized identically to the ZIF-8 material as described above, replacing zinc chloride with the equivalent quantity of cobalt chloride hexahydrate. A scheme of the synthesis paths is described in Figure 1 [39,40]. 

### 2.3. Synthesis of Gold Nanoparticles

Gold nanoparticles (AuNP) were prepared following procedure described in reference [41]. An aqueous solution of HAuCl_4_ (0.001 M, 40 mL) was placed into a 250 mL round bottom flask. The solution was heated to 90 °C followed by the addition of sodium citrate aqueous solution (38.8 mM, 2 mL) into it while stirring for 15 min. After cooling down to room temperature, the solution was centrifuged three times with ethanol and three times with DI water, finally the precipitate was re-dispersed in DI water, resulting a water solution with AuNP of ~5 nm.

### 2.4. Zeolitic Imidazolate Framework Nanocrystal Characterization

The synthetized samples were kept at room conditions and characterized using: Fourier transform-infrared spectroscopy (FTIR), X-ray diffraction (XRD), scanning electron microscope (SEM) and energy-dispersive X-ray (EDS). FTIR spectra were recorded using a Thermo Nicolet NEXUS 670 FTIR spectrometer. The sample was diluted into KBr pellets in a 1:100 weight ratio (sample to KBr). The scanning range was 400–4000 cm^−1^ and the resolution was 4 cm^−1^. XRD powder patterns were recorded with CuKα radiation in a D8 advance diffractometer from Bruker. The morphological features were examined by SEM. The SEM and EDS analysis were performed on a JEOL JMS-7600F.

### 2.5. Love-Wave Sensor

**Figure 2:**
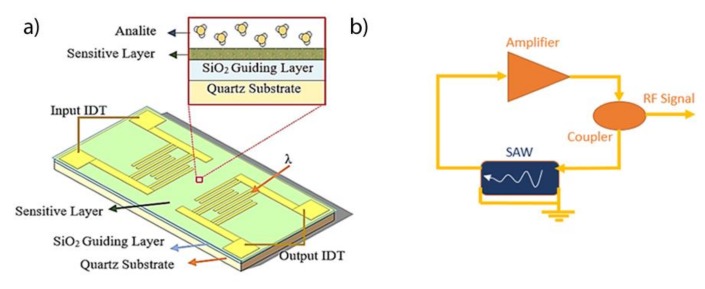
(**a**) Scheme representation of LW sensor and layer composition. (**b**) Oscillator circuit used to read the resonant frequency.

Love-waves (LW) are a specific type of SAW sensors based on shear horizontal (SH) waves guided by a layer with a lower propagation velocity than that of the piezoelectric substrate. The energy of the wave is confined in the guiding layer and any perturbation in it affects the acoustic wave velocity. The LW sensors used in the present work were designed with a delay line (DL) configuration. This device is based on a micro-electromechanical system composed of a piezoelectric material (ST-Quartz) with facing input/output aluminum interdigital transducers (IDT) on its surface, working at a 28 μm wavelength (λ), with a separation between IDTs of 2100 μm (Figure 2a). The SH waves were guided by a 3.1 μm thick layer of SiO_2_, with the sensitive layer at its surface. An oscillator circuit consisting of a DL, with an amplification stage and a coupler were used for measuring the changes in the velocity of the waves by means of the resonant frequency (Figure 2b).

### 2.6. ZIF as Sensitive Layers

**Figure 3:**
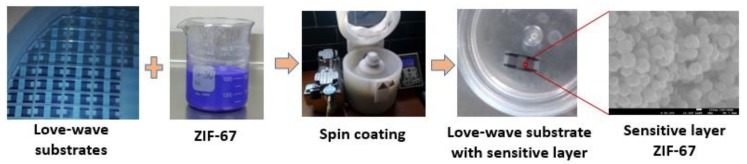
Process sequence of sensitive layer deposition on LW devices using the spin coating technique.

The eNose was based on a SAW sensor array with different sensitive layers to achieve a specific fingerprint for analytes of interest. The sensitive material samples were obtained by mixing each main solution with a volumetric proportion of 75% (solution-1) and 25% (solution-2) (Table 1). Spin coating was used to deposit a thin layer of the sensitive material. The process consisted of putting 25 µL of sample directly on the SiO_2_ guiding layer, completely covering the IDTs surface and the area between them, and then spinning the assemblage at a speed of 3000 rpm for one minute, ensuring that the sensing area remained constant from sensor to sensor (Figure 3). A suitable thickness for each sensor is obtained after depositing four times in a multilayer configuration achieving an optimal sensitivity for sensors. After ZIFs deposition process a thermal treatment at 180 °C with 50 mL/min nitrogen flow in a tubular oven was applied during four hours for extracting the excess of the 2MeIM organic ligand in the sensitive layer.

### 2.7. Experimental Setup

**Figure 4:**
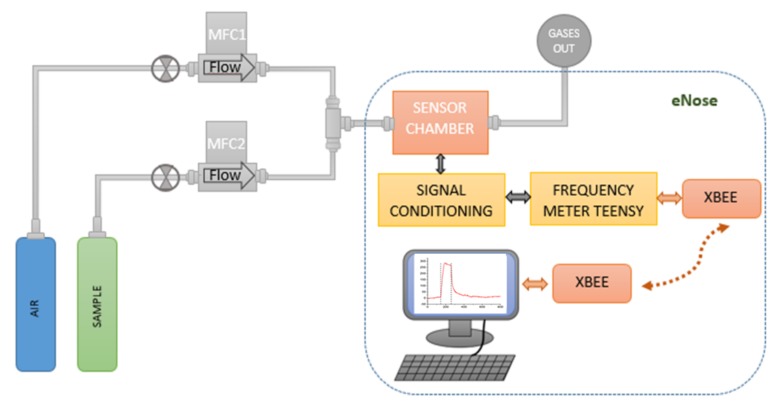
Experimental setup for the eNose characterization.

The sensor array was tested for acetone, ethanol, and ammonia gas analytes diluted in synthetic dry air to obtain concentrations of: 5 ppm, 10 ppm, and 25 ppm. The gas sample generator (Figure 4) consists of two mass flow controllers which were used to obtaining concentrations at a constant flow of 100 ml/min. Each array sensor works in an oscillator circuit, which includes an amplifier and a directional coupler. Therefore, when the sensor is perturbed, the oscillating frequency is shifted. A heterodyne configuration was used for signal acquisition, mixing the signal of the oscillator coupled to a reference sensor of the array (local oscillator) with the signal from an oscillator coupled to a selected ZIF nanocrystal-based SAW sensor (input signal). The frequencies obtained from the mixer were lower than 1 MHz and were acquired by a microcontroller programed as a frequency counter. For the detection, a sensing system composed of a measuring instrument (eNose) manufactured to be operated with a SAW sensor array was used. Finally, the sensor array response was acquired by a micro-frequency counter, and the information was transmitted wirelessly by a radio module to a PC with a custom application developed to display and store experimental the sensor data in real time.

## 3. Results and Discussion

### 3.1. Structural and Morphological Characterization of ZIFs

**Figure 5:**
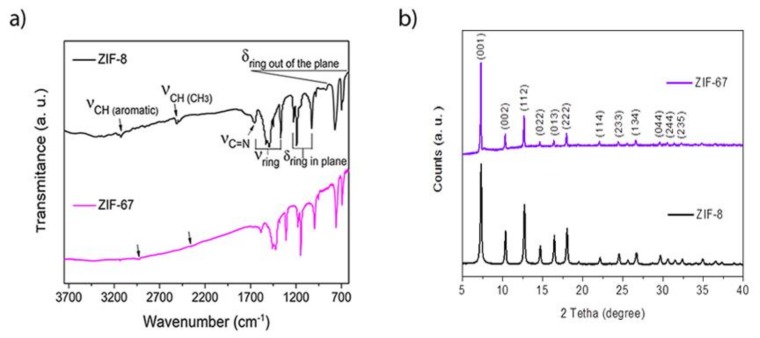
(**a**) FTIR spectra of the ZIF-8 and ZIF-67 samples diluted in KBr. (**b**) 2- XRD Powder Pattern of the ZIF-8 and ZIF-67 samples.

The FTIR spectra of KBr diluted ZIF-8 and ZIF-67 samples show the characteristic adsorption bands of 2meImidazole ring vibrations, reported for these structures in previous works [42] (Figure 5a). The XRD patterns of both ZIFs samples show evidence for the formation of a largely crystalline structure with long-range order (Figure 5b). The position and relative intensity of the diffraction maxima are in agreement with the literature for ZIF-8 and ZIF-67 frameworks [38,39,42].

**Figure 6:**
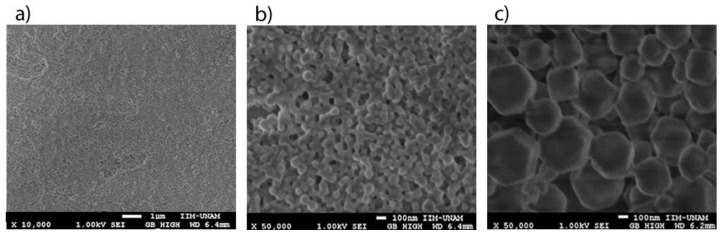
SEM image with magnification of (**a**) 10,000× for a continuous layer of ZIF-8 nanocrystals, (**b**) 50,000× for a layer of ZIF-8 nanocrystals and (**c**) 50,000× for a layer of ZIF-67 nanocrystals.

SEM images from the ZIFs layers show that the nanocrystals were configured as a continuous layer with very small particles (Figure 6a). This fact is important because nanostructured layers have two main advantages: first, the surface area of interaction with the gaseous environment is higher; and second, the SAW propagates in a continuous layer with very low scattering losses because to its wavelength (28 μm) is much larger than the diameter of the nanocrystals. The micrographs also showed hexagonal shaped nanocrystals of 50 nm approximately for the ZIF-8 samples (Figure 6b) and 200 nm approximately for the ZIF-67 samples (Figure 6c). 

### 3.2. Electrical Characterization of the Love-Wave Sensors

**Figure 7:**
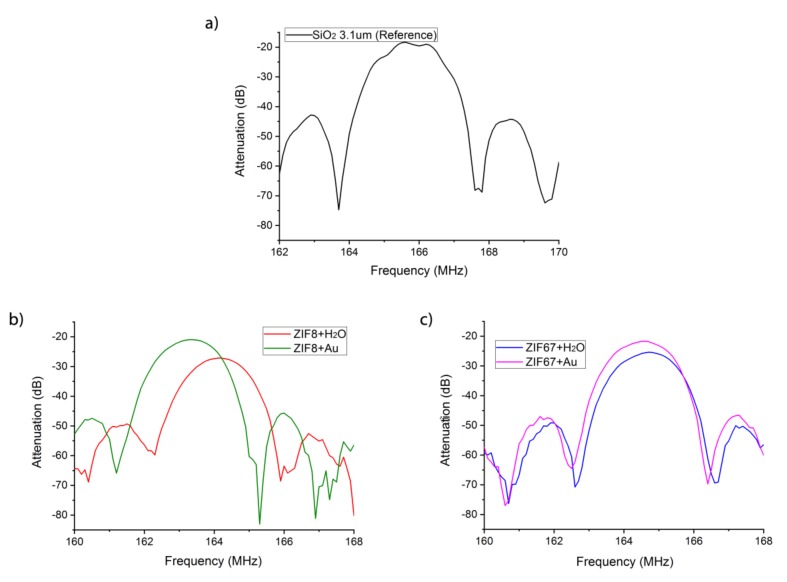
Spectral response of the LW sensors (**a**) without (**b**) with ZIF8 and (**c**) with ZIF67 layers.

The LW sensors were characterized before and after depositing the ZIF nanocrystal sensitive layers. In the array, a reference device without a sensitive layer was used (Figure 7a) to compensate sensor responses for undesirable changes in temperature and pressure. The sensors were characterized by means of the automatic network analyzer (ANA Wiltron 360B, WILTRON CO., Ltd., Incheon, Korea) and the S21 parameter was used to measure insertion loss transmission. The frequency response of each sensor exhibited a frequency shift of the minimum insertion loss caused by the mass loading of the sensitive material (Figure 7b,c). On the other hand, increases of insertion losses (Table 2) were a consequence of the scattering due to the propagation of the SAW wave in the sensitive material.

### 3.3. Gas Characterization

**Figure 8:**
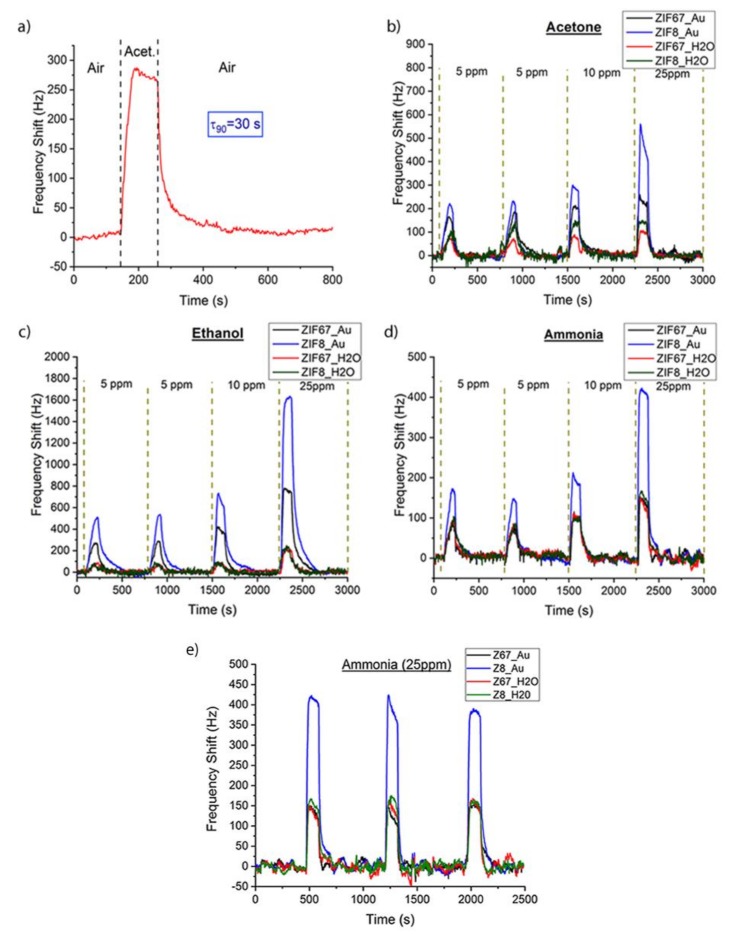
(**a**) SAW eNose response to 10 ppm of acetone. Experimental response for 5 ppm, 10 ppm and 25 ppm of (**b**) acetone, (**c**) ethanol and (**d**) ammonia with S1, S2, S3, and S4 sensitive layers. (**e**) Three consecutive measurements for 25 ppm of ammonia.

The SAW eNose based on a LW sensor array with ZIFs nanocrystals was tested with acetone, ethanol and ammonia markers related to diabetes mellitus disease. The sensors were exposed for two minutes to each analyte at concentrations of 5 ppm, 10 ppm and 25 ppm, and then the array was purged with dry synthetic air for 10 min. The LW sensors showed a notable and fast response, e.g., for 10 ppm of acetone a frequency shift of 275 Hz and a τ_90_, around 30 s, with a complete recovery achieved after 10 min (Figure 8a), τ_90_ being defined as the time taken to reach 90% of the frequency shift. The response of the sensor array to different concentrations of acetone (Figure 8b), ethanol (Figure 8c), and ammonia (Figure 8d) showed a high frequency shift for the different sensitive layers tested, obtaining best sensitivities for S_2_ (ZIF8_Au). The measurement reproducibility was tested in two different forms; first, the lowest concentration (5 ppm) was measured twice in continuous cycles, during which a similar frequency shift was obtained; and second, the measurement with 25 ppm of ammonia was repeated three times, as a control test (Figure 8e), showing similar values of frequency shifts. Therefore, the eNose can be used for measurements in a few seconds and repetitions or new measurements can be carried out after ten minutes.

**Figure 9:**
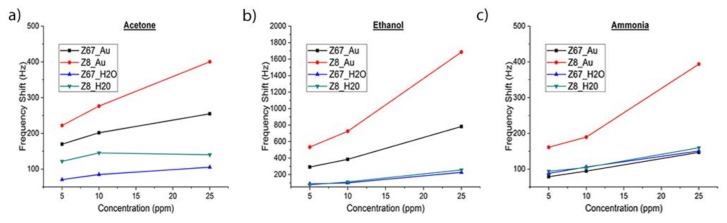
Calibration curves of the sensors S1, S2, S3 and S4 for (**a**) acetone, (**b**) ethanol and (**c**) ammonia.

The reference sensor helped to compensate in the sensor array the pressure and temperature changes due to external factors, measuring only variations related to the interaction of the analytes with the sensitive layers (Table 3). The frequency shifts at the end of the exposure time were taken as responses and calibration curves were obtained (Figure 9). The responses of the sensors were found to increase with higher concentrations.

The ZIFs/AuNP layers deposited on SiO_2_ showed better mechanical properties than the ZIFs layers. In spite of a larger shift of the SAW response towards a lower frequency, they had lower insertion losses, which explains the enhanced gas-sensing properties of ZIFs/AuNP layers for the three samples. However, in the cases of acetone and ethanol, the ZIFs/AuNP layers had a significantly increased sensitivity that can be attributed to the strong interaction Au-gas molecule [43]. On the other hand, the greater sensitivity of the ZIFs/AuNP layers for ethanol can be associated with the well-known catalytic activity of <5 nm sized AuNPs for selective oxidation of alcohols [44]. Ethanol adsorbed on the AuNPs surface can then be oxidized, even at room conditions, to acetaldehyde which is detected with enhanced sensitivity by the ZIFs [21,45].

**Figure 10:**
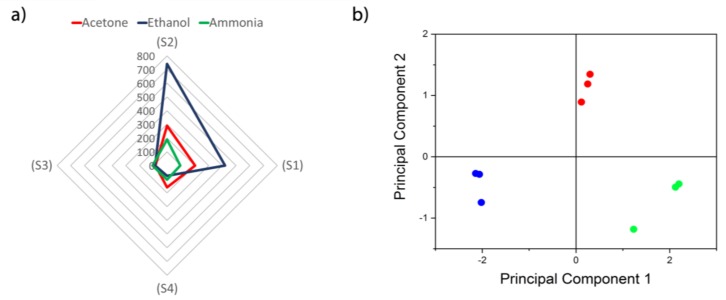
(**a**) Radial representation of the sensor array responses to 10 ppm of acetone, ethanol and ammonia. (**b**) Principal components analysis applied to data for discrimination of acetone (red), ethanol (blue) and ammonia (green).

The present sensor array with nanocrystalline ZIFs as sensitive materials allows detection and discrimination of acetone, ammonia, and ethanol, as sensor responses show in the radial surface analysis to 10 ppm of the three markers (Figure 10a). In order to make discrimination and classification more feasible in a real case, for a given marker concertation the response for each sensor was normalized to the sum of the responses of the different sensors. By doing so, the information in the data of all sensors is conserved. Principal component analysis (PCA) was applied to this data and a total separation is observed among the markers (Figure 10b). This shows that the SAW eNose presented can be used to relate specific fingerprints to diabetes.

The present sensors have some significant advantages over the chemoresistive sensors based on ZIFs. First, SAW sensors show high sensitivity towards acetone, ethanol and ammonia at room temperature and chemoresistive sensors possess good sensitivity at high temperatures but poor sensitivity at room temperature [21,38,45]. Second, ZIF-8 has a high electrical resistance, requiring its combination with other materials to develop chemoresistive sensors [21,38]. Finally, a chemoresistive sensor based on ZnO nanorods showed gas selective of gases when combined with ZIF–8 shell [21], decreasing sensitivity towards acetone, ethanol or ammonia; however, SAW devices with ZIF sensitive layers combined with nanoparticles improve the sensitivity towards acetone and ethanol.

## 4. Conclusions

A SAW eNose based on Love-wave sensors combined with ZIF-8, ZIF-67, ZIF-8/AuNP and ZIF-67/AuNP as sensitive layers was tested to three breath markers of Diabetes mellitus: acetone, ethanol and ammonia at concentrations of 5 ppm, 10 ppm and 25 ppm. 

It has been shown that the SAW/ZIF eNose is effective in obtaining high sensitivity, selectivity, and reproducibility. Fast detection and recovery responses have been achieved as well, detecting concentrations as low as 5 ppm of ammonia. Finally, the efficiency of the system to discriminate Diabetes mellitus markers has been demonstrated using principal component analysis. 

In conclusion, tests carried out in this work exhibited a properly performance of the SAW/ZIF eNose to be proved in future works as a prototype for a noninvasive system contributing to the diagnosis and control of Diabetes mellitus in real cases.

## Figures and Tables

**Table 1:** Different composition of the sensitive layers used in the SAW sensors included in the eNose.

Samples	Solution-1 (750 µL)	Solution-2 (250 µL)
S1	ZIF-67	Au-NPs
S2	ZIF-8	Au-NPs
S3	ZIF-67	H_2_O-DI
S4	ZIF-8	H_2_O-DI

**Table 2:** Insertion loss and frequency shift with respect to the reference sensor, 165 MHz and 18.3 dB.

Sensors	Sensitive Layer	Attenuation (dB)	Frequency Shift (Hz)
S1	ZIF-67 + AuNPs	3.4	1100
S2	ZIF-8 + AuNPs	2.6	2400
S3	ZIF-67 + H2O	7.1	900
S4	ZIF-8 + H2O	8.7	1500

**Table 3:** Sensitivity, limit of detection and response time of the sensors S1, S2, S3 and S4 obtained from responses of 10 ppm.

Sensor	Sensitivity (Hz/ppm)			Limit of Detection (ppm)			Response Time (s)		
	Acet.	Etha.	Ammo.	Acet.	Etha.	Ammo.	Acet.	Etha.	Ammo.
S1	20	38	9	1.5	0.8	3.2	33	30	24
S2	28	72	19	1.1	0.5	1.6	30	40	27
S3	8	10	11	3.6	3.0	2.9	36	38	38
S4	15	11	10	2.1	2.8	2.9	44	36	41
